# Clinical outcomes of 77 TESE treatment cycles in non-mosaic Klinefelter syndrome patients

**DOI:** 10.5935/1518-0557.20210081

**Published:** 2022

**Authors:** Pedro Barros, Mariana Cunha, Alberto Barros, Mário Sousa, Sofia Dória

**Affiliations:** 1 Department of Genetics, Faculty of Medicine, University of Porto, Porto, Portugal; 2 Centre for Reproductive Genetics A. Barros, Porto, Portugal; 3 Institute of Health Research and Innovation (IPATIMUP/i3S), University of Porto, Porto, Portugal; 4 Laboratory of Cell Biology, Department of Microscopy, Institute of Biomedical Sciences Abel Salazar (ICBAS), University of Porto, Porto, Portugal; 5 Unit for Multidisciplinary Investigation in Biomedicine, ICBAS-UP, Porto, Portugal

**Keywords:** azoospermia, clinical outcomes, non-mosaic Klinefelter syndrome, testicular spermatozoa extraction, newborn

## Abstract

**Objective:**

The current study aimed to present the clinical outcomes of 76 azoospermic patients with non-mosaic Klinefelter syndrome (KS), treated with testicular spermatozoa extraction (TESE) followed by intracytoplasmic sperm injection (ICSI) using either fresh or cryopreserved testicular spermatozoa.

**Methods:**

We retrospectively evaluated 76 patients with non-mosaic KS belonging to a special group of cases that besides infertility did not present the classical signs and symptoms of testosterone deficiency. One of the patients repeated the TESE procedure (76 patients, 77 TESE cycles). Sixty of these 76 patients accepted to undergo TESE associated with ovarian stimulation, while 16 patients underwent TESE followed by testicular spermatozoa cryopreservation. Aneuploidy screening of the offspring was performed by Multiplex ligation-dependent probe amplification and by amniotic fluid karyotyping. Statistical analysis used the Chi-Squared Test, Fisher's Exact Test, 2-sided, for rates, and the Independent Samples T-test for equality of means, 2-sided.

**Results:**

Testicular spermatozoa were recovered in 31 (40.3%) of the attempts. The patients underwent 47 ICSI cycles, 25 with fresh testicular spermatozoa and 22 with cryopreserved testicular spermatozoa. Fertilization (63.5% vs. 41.6%, p=0.000), implantation (37% vs. 13.2%, p=0.014), clinical pregnancy (60.9% vs. 19%, p=0.005) and live birth (65.2% vs. 23.8%, p=0.006) rates were higher with fresh testicular spermatozoa. Chromosome analysis of the 21 newborns was normal.

**Conclusions:**

The present data adds further information regarding the recovery rate of spermatozoa after TESE and the embryological and clinical outcomes with fresh and cryopreserved testicular spermatozoa, besides reassuring the safety concerning chromosomal transmission of KS from parents to their offspring.

## INTRODUCTION

Klinefelter syndrome (KS) is a male hypergonadotropic hypogonadism (Klinefelter *et al*., 1942) caused by the presence of an extra X chromosome (Jacobs & Strong, 1959). The classical phenotype is characterized by small firm testes, gynecomastia, eunuchoid body proportions, high levels of gonadotropins (FSH, LH), low/normal levels of testosterone, and progressive testicular insufficiency causing androgen deficiency (Groth *et al*., 2013). The syndrome affects about 0.15% of male newborns in the general population (Gravholt *et al*., 2018). The most common karyotype is 47,XXY (80-90% of the cases), being the mosaic form (47,XXY in combination with a normal 46,XY line or other aneuploid lines) less frequent (10-20% of the cases) (Lanfranco *et al*., 2004), with the supernumerary X chromosome derived from male or female nondisjunction errors at the first meiotic division (Thomas & Hassold, 2003). In KS patients, hormone levels and testicular morphology vary from the fetal period to adulthood. Gradual increases in FSH and LH levels have been observed, along with decreases in Inhibin and Anti-Müllerian hormone (AMH) levels, maintenance of testosterone and Insulin-like factor-3 (INSL3) levels (Aksglaede *et al*., 2006), a gradual loss of spermatogonia with loss of functional seminiferous tubules, fibrosis and hyalinization of the interstitium, and Leydig cell hyperplasia (Wikström & Dunkel, 2008; Van Saen *et al*., 2018).

Patients with KS present a broad spectrum of phenotypes, with different severity degrees of androgen deficiency, mainly because of the non-inactivation of several critical X-linked genes (Bonomi *et al*., 2017). This enables the existence of testicular foci with preserved spermatogenesis. In these cases, cytogenetic studies revealed the presence of spermatogonia lines with a 47,XXY and/or 46,XY chromosome constitution, with the presence of haploid spermatozoa derived from both spermatogonia lines (Sciurano *et al*., 2009; Garcia-Quevedo *et al*., 2011; Miki *et al*., 2017). Testicular sperm extraction (TESE) applied to KS patients revealed foci of spermatogenesis in about 40% of the cases, with testicular sperm then used in infertility treatments (Tournaye *et al*., 1996; Palermo *et al*., 1998).

Only three studies have reported on a large number of KS patients whose infertility treatments included cryopreserved testicular spermatozoa (Greco *et al*., 2013; Madureira *et al*., 2014; Vicdan *et al*., 2016). In the present report, we evaluated 76 azoospermic patients with non-mosaic KS that accepted to undergo treatment with TESE-ICSI. All patients belong to a group of cases referred only due to infertility, without classical signs and symptoms of testosterone deficiency. Patient characteristics, testicular sperm retrieval rates and clinical outcomes after treatment with fresh and frozen-thawed testicular spermatozoa were analyzed. There were 21 newborns, all healthy and without chromosomal abnormalities.

## MATERIALS AND METHODS

### Ethics guidelines

This study did not involve experimentation with humans or animals. Patient data were used only with a statistical purpose. According to the National Law on Medically Assisted Procreation (Law 32/2006) and the National Council for Medically Assisted Procreation guidelines (2018), the use of clinical databases for research is allowed without need for approval by an Ethics Committee, as long as the data is anonymized and patients give consent in written. Data collection followed the principles outlined in the Declaration of Helsinki. All men previously gave informed consent agreeing to share their anonymized information for future studies. The University Hospital Ethics Committee approved the study and assigned it certificate number 2019/CE/P017 (266/CETI/ICBAS). Patients were informed of the technical possibility of preimplantation genetic testing for aneuploidy and of prenatal diagnosis.

### Patients

We retrospectively evaluated 139 patients with azoospermia due to non-mosaic KS seen in consecutive years (1994-2019). These patients belong to a special category of KS, since all went to private IVF clinics due to infertility in adult age, without showing the classical signs and symptoms of testosterone deficiency. None of the patients had ever received hormone replacement therapy. The same urologist evaluated all KS patients and performed the testicular biopsies. The genetic analysis of KS patients and their offspring was performed at a University Department of Genetics. Clinical diagnosis was based on the absence of sperm in semen, confirmed after centrifugation, decreased testicular volume and increased serum levels of FSH and LH, and karyotype confirmation of the 47,XXY status.

Karyotypes were obtained using G banding, with analysis of at least 30 metaphases from peripheral blood lymphocytes in accordance to general protocols (Rooney & Czepulkowski, 1997). Aneuploidy screening of the offspring was performed in 18 cases by Multiplex ligation-dependent probe amplification (MLPA) for chromosomes 13, 18, 21, X and Y, using oral epithelial cells obtained at the postnatal period in 17 cases and DNA extracted from paraffin blocks in one case of a neonatal death. In the remaining 3 cases, prenatal diagnosis was performed and karyotypes were obtained from amniotic fluid. DNA extraction from oral epithelial cells was performed using a commercial kit (JETQUICK, Blood & Cell Culture DNASpin Kit; Genomed, Lohne, Germany). DNA extraction from paraffin-embedded material was performed using the DNeasy Blood and Tissue Kit (QIAGEN, Hilden, Germany). Aneuploidy screening was performed using the MLPA technique (Kit SALSA P095; MRC-Holland, Amsterdam, The Netherlands) (Schouten *et al*., 2002).

The volume of each testis was determined by two sequential methods, one using the Prader orchidometer (12 ellipsoid solid models of 1-25 ml) and the other an orchidometer with ellipsoid rings of 1-30 ml. The testicular volumes were considered atrophic when the volume was <6ml, hypotrophic when between 6-15 ml, and normal when >15ml (Bujan *et al*., 1989).

Of the 139 patients, 76 accepted to undergo treatment by TESE, and one repeated the TESE procedure (76 patients, 77 TESE cycles). Sixty of these 76 patients accepted to undergo TESE associated with ovarian stimulation, once they planned to use donor sperm in case of testicular sperm retrieval failure. The other 16 patients underwent diagnostic TESE followed by testicular sperm cryopreservation. The latter cases preferred to postpone ovarian stimulation, as they did not intend to use donor sperm in case of testicular sperm retrieval failure. The outcomes of these 76 patients were the object of the present study.

### Testicular biopsy

We performed open testicular biopsies by testicular spermatozoa extraction (TESE), where a single small surgical window allows the collection of testicular tissue at different levels. A microscope was not needed in the operating room. We used a local nerve anesthetic protocol (Sousa *et al*., 2002; Madureira *et al*., 2014). Spermatic cord block was performed according to the three-finger technique (Li *et al*., 1992; Gorgy *et al*., 1998; Nudell *et al*., 1998). Local anesthesia was achieved with 5-10 ml of a 1:1 mixture of 1% lidocaine hydrochloride solution (Xylocaine 2% without epinephrine; rapid action) and 0.5% bupivacaine (Marcaine 0.5% without epinephrine; long action). More recently, we have used 10% ropivacaine instead of bupivacaine, since it has a longer action period, enabling local discomfort relief for 2-3 h. In cases where the patient was more tolerant, anesthesia was performed only with 10% ropivacaine.

After a few minutes (about 5 min), a skin weal was raised in the scrotum adjacent to the middle region of the testis. A 1 cm transverse incision was then made and the tunica vaginalis space was entered. An incision of 0.5 cm then enabled the excision of a small piece of the seminiferous tubules (1-2 mm). A preliminary microscopic check of the sample (observation in an inverted research microscope, equipped with Hoffman optics and operated at room temperature) at the end of each biopsy avoided unnecessary tissue sampling. For this, each fragment was collected in Sperm Preparation Medium (SPM-Hepes; Origio, Jyllinge, Denmark) and then gently fragmented and squeezed to release luminal epithelial cells. Whenever needed and possible, the contralateral testis was biopsied. At the end of the biopsy procedure, and after careful cleaning and hemostasis, the tunica albuginea, the vaginal, the scrotum layers and the skin were closed. The procedure took about 30-45 minutes and was performed entirely on an outpatient basis. The procedure enabled rapid recovery with minimal complains and total absence of surgical complications, and none of the patients required hormone replacement therapy postoperatively. Oral tramadol and paracetamol were given to relieve discomfort in the first 24 h. When another biopsy was needed (one case), the procedure was scheduled to six months later to enable local repair and reestablishment of the vascular supply (Schlegel & Su, 1997).

The testicle with the highest volume was biopsied first. Testicular biopsy was mainly unilateral (87% of the cases, 67/77) and was performed on the right testicle in 68.8% (53/77) of the cases, on the left testicle in 18.2% (14/77) of the cases, and bilaterally in 13% (10/77) of the cases.

In cases with successful spermatozoa retrieval, testicular biopsy was unilateral in 96.8% (30/31) of the cases. The procedure was performed on the right testicle in 64.5% (20/31) of the cases, on the left testicle in 32.3% (10/31) of the cases, and bilaterally in 3.2% (1/31) of the cases.

In cases without successful spermatozoa retrieval, testicular biopsy was unilateral in 80.4% (37/46) of the cases. Biopsy was performed on the right testicle in 71.7% (33/46) of the cases, on the left testicle in 8.7% (4/46) of the cases, and bilaterally in 19.6% (9/46) of the cases.

At the laboratory, seminiferous tubule fragments were sorely mechanically fragmented and squeezed under a heated stereomicroscope to release luminal epithelial cells (Verheyen *et al*., 1995). If a good number of spermatozoa was obtained, the fluid fraction resulting from mechanical dissection was used for spermatozoa selection to ICSI. If the sample was contaminated with erythrocytes, the fluid fraction resulting from mechanical dissection was washed in SPM (2 x 5 min, 300 g) and the resulting cell pellet incubated for 5 min (37ºC, 5% CO_2_, filtrated humidified air) in 2 ml erythrocyte-lysing buffer [155 mM NH_4_Cl, 10 mM KHCO_3_, 2 mM EDTA, in water, pH 7.2 with KOH, 0.2 µm filtered (Millipore); endotoxin free, embryo and cell culture tested; Sigma, Barcelona, Spain)], followed by the addition of 2 ml SPM. The cell suspension was then washed in SPM (2 x 5 min, 300 g) and the cell pellet resuspended in SPM (Verheyen *et al*., 1995). Mechanical fragmentation and squeezing, and the use of the erythrocyte-lysing buffer were previously shown to enhance the efficiency of sperm selection without affecting sperm vitality or embryological and clinical outcomes (Verheyen *et al.,* 1995; Nagy *et al*., 1997).

When a lower number of spermatozoa was retrieved, the fluid fraction resulting from mechanical dissection was treated with erythrocyte-lysing buffer and enzymatically digested. For tissue digestion, the resulting cell pellet was washed in SPM (2 x 5 min, 500 g) and the pellet digested for 20 min [25 µg crude DNase, 1000 units collagenase-IV (Sigma) in 1 ml SPM] in the incubator (37ºC, 5% CO_2_, filtrated humidified air). Cells in the suspension were then separated (5 min, 50 g) into supernatant (mainly haploid germ cells) and pellet (mainly diploid germ cells and a few Sertoli cells) fractions. The fractions were then washed with SPM (2 x 5 min, 1000 g), and the final pellets were resuspended in 100 µl of SPM, followed by incubation at 32ºC until use.

Observations were performed on the thermal stage (32ºC) of an inverted microscope (Nikon DIAPHOT 200; Nikon, Tokyo, Japan) equipped with Hoffman optics (Nikon) and Narishige micromanipulators (MO-188; Narishige, Tokyo, Japan). To search for spermatozoa, 10-20 µl of the cell suspension were diluted in a 20-30 µl SPM microdrop placed in a sterile cell culture plastic Petri dish (60 mm, Falcon), under paraffin oil (Origio), which was then spread to facilitate the search. When spermatozoa were found, they were transferred to another SPM microdrop using microinjection needles (0.009 mm; Swemed, Goteborg, Sweden). In difficult cases, the search for motile spermatozoa took several hours. Enzymatic digestion followed published protocols, which also showed to enhance the efficiency of spermatozoa selection while not affecting spermatozoa vitality or embryological and clinical outcomes (Crabbé *et al*., 1997; 1998).

### Testicular sperm cryopreservation and thawing

In cases with a good number of surplus spermatozoa, the fluid was cryopreserved. In cases with low spermatozoa numbers, the fluid was enzymatically digested and the two fractions, supernatant and resuspended pellet, were cryopreserved separately. In cases with only a few surplus spermatozoa, cryopreservation was not performed. Testicular sperm suspensions (250 µl) were mixed to a 1:1 dilution with 250 µl of Sperm-Freezing-Medium or Cryosperm (Måløv, Denmark) and left to equilibrate for 10 min at room temperature. The cell suspensions were then aspirated into labeled straws, sealed (L`Aigle, France), left over LN2 vapor (30 min), and then immersed and stored in LN2. For thawing, straws were left for 15 min at room temperature and then washed with SPM (2 x 10 min, 500 g). The pellet was then resuspended in 50-100 µl of SPM and an extensive search for motile spermatozoa followed.

### Ovarian stimulation

Women underwent controlled ovarian hyperstimulation with a GnRH antagonist protocol in the majority of the cases (Merck Serono, Geneve, Switzerland; Organon, Oss, Netherlands) and with an agonist protocol in the remaining cases (Sanofi Aventis, Frankfurt, Germany). For stimulation, recombinant follicle stimulating hormone (rFSH) was used in most cases (Organon; Merck Serono), while human menopausal gonadotropin (HMG) was used as an alternative (Ferring, Kiel, Germany). Ovulation trigger was performed either with human chorionic gonadotropin (Organon) or recombinant HCG (Merck-Serono). Estradiol serum levels were analyzed on the day of HCG or one day before (Pinto *et al*., 2009).

### Gamete and embryo handling

Gametes and embryos were handled with media from Origio (Jyllinge, Denmark) or Vitrolife (Kungsbacka, Sweden). Microinjection was performed in an inverted microscope (Nikon DIAPHOT 200; Nikon, Tokyo, Japan) with Narishige micromanipulators (MO-188; Narishige, Tokyo, Japan), using micropipettes from Swemed (Goteborg, Sweden). ICSI was performed using strong dislocation of the cytoplasm (Tesarik & Sousa, 1995). Cleavage embryos (Vandervorst *et al*., 1998) and blastocysts (Gardner *et al*., 2000) were graded based on the Istanbul consensus (Alpha Scientists in Reproductive Medicine and ESHRE Special Interest Group of Embryology, 2011). Ultrasound guided embryo transfer was performed with a Sure View Wallace Embryo Replacement Catheter or Wallace malleable stylet (Smiths Medical Int, Kent, UK).

### Luteal supplementation

Luteal supplementation was accomplished with intravaginal administration of 600 mg daily (200 mg, tid) of natural micronized progesterone (Jaba, Besins Int, Montrouge, France) beginning on the day of oocyte retrieval. Implantation was confirmed by a rise in serum βHCG 12 days after embryo transfer. Clinical pregnancy was established by ultrasound at 6 weeks of gestation. Progesterone was maintained until βHCG serum assay and, if positive, it was continued until 8 weeks of gestation.

### Statistical analysis

Statistical analysis was performed with the IBM SPSS Statistics 20 program for Windows, using the Chi-Squared Tests, Fisher's Exact Test, 2-sided and the Independent Samples T-test for equality of means, 2-sided. A value of *p*<0.05 was considered statistically significant.

## RESULTS

### Testicular biopsy and patient characteristics

One of the 76 patients with non-mosaic KS repeated the TESE procedure. Testicular spermatozoa were recovered in 31/77 (40.3%) of the cases. TESE was performed in 61 of the 77 cases (25/61, 41% of sperm retrieval rate) in stimulated cycles, since couples accepted to use donor sperm if sperm retrieval on TESE failed; and 16 cases performed diagnostic TESE (6/16, 37.5% of sperm retrieval rate) outside a stimulated cycle with couples who refused to use donor sperm. In the latter cases, spermatozoa were cryopreserved. Patients unable to retrieve spermatozoa decided either for no treatment (9 cases) or to use donor spermatozoa (37 cases). Three of the 46 cases in which spermatozoa retrieval was unsuccessful presented maturation arrest at the primary spermatocyte stage and 43 had Sertoli cell only syndrome.

Comparisons between the 31 cases of successful spermatozoa retrieval and the 46 cases of failed TESE revealed no significant differences regarding male partner mean age (*p*=0.356), time of infertility (*p*=0.706), testicular volume (*p*=0.479), serum levels of FSH (*p*=0.660), LH (*p*=0.362) and testosterone (p=0.089), total number of testicular fragments (*p*=0.596) analyzed, or time of search (*p*=0.138) in samples (Table 1). Male partner mean age was 34.1±4.1 (range: 26-46) years. In cases resulting in live births, male partner mean age was 33.6±3.0 (28-38) years.

**Table 1.** Clinical data of patients with non-mosaic Klinefelter syndrome submitted to TESE.

Parameters	TESE with sperm retrieval	TESE without sperm retrieval	*p* value a	Total
TESE (n)	31	46		77
Male age (years)	33.5±3.8 (28-46)	34.4±4.3 (26-42)	0.356	34.1±4.1 (26-46)
Time of infertility (years)	3.8±2.4 (1-10)	4.1±3.6 (1-17)	0.706	4.0±3.2 (1-17)
Total testicular volume (ml)	7.3±3.7 (3-16)	8.0±4.4 (3-16)	0.479	7.7±4.2 (3-16)
FSH (mIU/ml)	29.6±16.2 (11.8-62.2)	31.1±9.8 (10.4-54.2)	0.660	30.5±12.6 (10.4-62.2)
LH (mIU/ml)	16.6±5.9 (4.4-29.4)	18.5±8.3 (1.9-50)	0.362	17.7±17.3 (1.9-50)
Testosterone (ng/ml)	7.0±4.8 (2.42-18)	10.7±9.0 (2.45-30.86)	0.089	9.1±7.6 (2.42-30.86)
Number of Fragments Biopsied Total Right Testicle Left Testicle	8.1±3.2 (2-18) 8.0±3.8 (2-18) 7.6±2.7 (3-12)	7.7±2.9 (3-15) 7.1±2.5 (2-13) 4.3±1.9 (1-8)	0.596 0.297 0.002	7.9±3.1 (2-18) 7.4±3.0 (2-18) 5.8±2.8 (1-12)
Time of Search	3.5±3.0 (0.5-16)	4.1±0.7 (1.5-5)	0.138	3.9±2.0 (0.5-16)

Values in: mean ± SD, (range).

TESE=testicular sperm extraction

n=number of TESE cases

FSH (normal range: 0.7-11.1 mIU/ml)

LH (normal range: 0.8-7.6 mIU/ml),

Testosterone (normal range: 2.45-16 ng/ml),

Significant differences between groups with and without sperm retrieval (*p*<0.05).

Testicular volume was decreased in 96.1% of all cases; 40.3% were atrophic, 55.8% were hypotrophic, and 3.9% were normal. Testicular volume was decreased in 96.8% of the cases with successful sperm retrieval; 38.7% were atrophic, 58.1% were hypotrophic, and 3.2% were normal. Testicular volume was decreased in 95.7% of the cases of failed TESE; 41.3% were atrophic, 54.4% were hypotrophic, and 4.4% were normal. Testicular volume was decreased in 92.3% of the cases that resulted in live births.

Mean serum FSH levels were increased in 98.3% of all cases; 1.7% had normal levels and none had decreased FSH levels. Mean FSH levels were increased in all cases (100%) with successful sperm retrieval. Mean FSH levels were increased in 97.2% of the cases of failed TESE; 2.8% had normal levels and none had decreased FSH levels. Mean FSH serum levels were increased in all cases that resulted in live births.

Mean serum LH levels were increased in 94.1% of all cases; 5.9% had normal levels and none had decreased LH levels. Mean LH serum levels were increased in 90.9% of the cases with successful sperm retrieval; 9.1% had normal levels and none had decreased LH levels. Mean LH serum levels were increased in 96.6% of the cases of failed TESE; 3.4% had normal levels and none had decreased LH levels. Mean LH serum levels were increased in 81.8% of the cases that resulted in live births.

Mean serum testosterone levels were normal in 77.6% of all cases; 20.4% had increased levels and 2% had decreased serum testosterone levels. In cases with successful TESE, the Mean serum testosterone levels were normal in 85.7% of the cases with successful TESE; 9.5% had increased levels and 4.8% had decreased serum testosterone levels. Mean serum testosterone levels were normal in 71.4% of the cases with failed TESE; 28.6% had increased levels and none had decreased testosterone levels. Mean serum testosterone levels were normal in 91.7% of the cases that resulted in live births. The patient with decreased testosterone levels presented borderline values (one TESE cycle, one female newborn).

### Embryological, clinical and newborn outcomes

A total of 47 ICSI treatment cycles were performed in the 31 patients with successful spermatozoa retrieval. Twenty-five cycles (23 with embryo transfer) used fresh testicular spermatozoa and 22 (21 with embryo transfer) used frozen-thawed testicular spermatozoa, adding up to 44 cycles with embryo transfer. Seventeen used cryopreserved spermatozoa from the surplus spermatozoa originated from TESE cases using fresh spermatozoa in the first attempt and five cases from diagnostic TESE. Three of the six cases with successful spermatozoa retrieval during diagnostic TESE had one treatment cycle with cryopreserved spermatozoa; one case had two cycles with cryopreserved spermatozoa; and two cases declined treatment, which yielded 22 (17 + 5) ICSI cycles with cryopreserved testicular spermatozoa.

In terms of embryological outcomes (Table 2), no significant differences between fresh and cryopreserved spermatozoa were found in relation to the mean number of cumulus-oocyte complexes (*p*=0.419) or mature oocytes (*p*=0.561) retrieved, or in oocyte maturation rate (80.3% *vs*. 82.2%, *p*=0.611). Cycles with fresh spermatozoa yielded a significantly greater mean number of fertilized oocytes (*p*=0.013) and higher fertilization rate (63.5% *vs*. 41.6%, *p*=0.000). Although the mean number of cleaved embryos was significantly higher in cycles with fresh spermatozoa (*p*=0.011), there were no significant differences in embryo cleavage rates (95.5% *vs*. 98.6%, *p*=0.256). Similarly, cycles with fresh spermatozoa showed a significantly greater mean number of day-3 embryos (*p*=0.015) and higher quality embryos at day-3 (*p*=0.011), but no significant differences were observed in relation to the rate of high quality embryos (81.7% *vs*. 77.6%, *p*=0.509). No significant differences were found in the mean number of blastocysts (*p*=0.200) or in blastocyst formation rates (50.8% *vs*. 56.5%, 0.641).

**Table 2.** ICSI embryological outcomes of patients with non-mosaic Klinefelter syndrome using fresh and frozen-thawed testicular spermatozoa.

Parameters	TESE/ICSI		*p* value	Total
	Fresh sperm	Cryopreserved sperm		
Cycles (n)	25	22	-	47
Embryo transfer cycles (n)	23	2	-	44
COC (n, mean ± SD, range)	259 (10.4±5.1) 2-19	202 (9.2±4.7) 2-19	0.419	461 (9.8±4.9) 2-19
MII (n, mean ± SD, range)	208 (8.3±5.0) 1-18	166 (7.5±3.9) 2-18	0.561	374 (8.0±4.5) 1-18
Maturity rate (%; MII/COC)	80.3% (208/259)	82.2% (166/202)	0.611	81.1% (374/461)
2PN/2PB (n, mean ± SD, range)	132 (5.3±3.5) 0-14	69 (3.1±1.8) 0-7	0.013	201 (4.3±3.0) 0-14
Fertilization rate (2PN/MII)	63.5% (132/208)	41.6% (69/166)	0.256	53.7% (201/374)
Cleaved embryos (n, mean ± SD, range)	126 (5.3±3.1) 1-14	68 (3.2±1.6) 1-6	0.011	194 (4.3±2.7) 1-14
Embryo cleavage rate (%, d2/2PN)	95.5 (126/132)	98.6 (68/69)	0.256	96.5 (194/201)
Day 3 embryos (n, mean ± SD, range)	126 (5.3±3.1) 1-14	58 (3.2±1.4) 1-6	0.015	184 (4.4±2.7) 1-14
Day 3 grade A/B embryos (n, mean ± SD, range)	103 (4.5±2.9) 1-13	45 (2.5±1.2) 1-6	0.011	148 (3.6±2.5) 1-13
Day 3 Grade A/B rate (AB/d3)	103/126 (81.7)	45/58 (77.6)	0.509	148/184 (80.4)
Day 5 embryos (n, mean ± SD, range)	40 (4.4±2.8) 2-9	13 (2.6±1.3) 2-5	0.200	53 (3.8±2.5) 2-9
Blastocyst rate (%, day 5/day 2)	50.8 (31/61)	56.6 (13/23)	0.641	52.4 (44/84)

TESE=testicular sperm extraction

ICSI=intracytoplasmic sperm injection

TESE/ICSI-fresh sperm=TESE/ICSI cycles using fresh testicular sperm

TESE/ICSI-cryopreserved sperm=TESE/ICSI cycles using cryopreserved testicular sperm

COC=cumulus-oocyte complexes

MII=mature oocytes

2PN/2PB=2 pronuclei and 2 polar bodies (normal fertilized oocytes)

D2=cleaved embryos at day 2.

Significant differences (*p*<0.05) between TESE/ICSI with fresh sperm and TESE/ICSI with cryopreserved sperm.

In relation to clinical outcomes (Table 3), there were no significant differences between fresh and cryopreserved spermatozoa regarding the mean number of transferred embryos (*p*=0.267); significant differences were observed in biochemical pregnancy (65.2% *vs*. 33.3%, *p*=0.035), clinical pregnancy (60.9% *vs*. 19%, *p*=0.005), implantation (37% *vs*. 13.2%, *p*=0.014), and live birth rates (52.2% *vs*. 19%, *p*=0.023). No significant differences were found in the rates of singletons (78.6% *vs*. 75%, *p*=0.880) or twins (21.4% *vs*. 25%, *p*=0.880). There were no ectopic pregnancies or stillbirths. Two singleton abortions occurred in cycles with fresh spermatozoa. Of the 44 embryo transfer cycles, 4 (9.1%) were performed on Day 2; 19 (43.2%) on Day 3; 8 (18.2%) on Day 4; and 13 (29.5%) on Day 5. The relative clinical pregnancy rates were 42.1% (8/19) on Day 3; 25% (2/8) on Day 4; and 61.5% (8/13) on Day 5; no pregnancies were achieved through embryo transfers on Day 2. The day of embryo transfer was generally similar between cycles with fresh and cryopreserved spermatozoa, as follows: Day 2 (4.3% *vs*. 14.3%); Day 3 (43.5% *vs*. 42.9%); Day 4 (17.4% *vs*. 19%); and Day 5 (34.8% *vs*. 23.8%). However, pregnancies rates were dissimilar: Day 2 (0% *vs*. 0%); Day 3 (70% *vs*. 11.1%); Day 4 (0% *vs*. 50%); and Day 5 (87.5% *vs*. 20%).

**Table 3.** ICSI clinical outcomes of patients with non-mosaic Klinefelter syndrome using fresh and frozen-thawed testicular spermatozoa.

Parameters	TESE/ICSI		*p* value	Total
	Fresh sperm	Cryopreserved sperm		
Cycles (n)	25	22	-	47
Embryo transfer cycles (n)	23	2	-	44
Nº of transferred embryos (n, mean ± SD, range)	46 (2.0±0.6) 1-3	38 (1.8±0.5) 1-3	0.267	84 (1.9±0.6) 1-3
Biochemical pregnancy (/ETC) (rate, n)	65.2% (15/23)	33.3 (7/21)	0.035	50 (22/44)
Clinical pregnancy (/ETC) (rate, n)	60.9 (14/23)	19 (4/21)	0.005	40.9 (18/44)
Sacs (n)	17	5	-	22
Implantation rate (nº sacs/nº ET)	37 (17/46)	13.2 (5/38)	0.014	26.2 (22/84)
Singletons (/CP) (rate, n)	78.6 (11/14)	75 (3/4)	0.880	77.8 (14/18)
Twins (/CP) (rate, n)	21.4 (3/14)	25% (1/4)	0.880	22.2 (4/18)
Abortion (/CP) (rate, n)	14.3 (2/14)	0	-	11.1 (2/18)
LBDR (/ETC) (rate, n)	52.2 (12/23)	19 (4/21)	0.023	36.4 (16/44)

TESE=testicular sperm extraction

ICSI=intracytoplasmic sperm injection,

TESE/ICSI-fresh sperm=TESE/ICSI cycles using fresh testicular sperm

TESE/ICSI-cryopreserved sperm=TESE/ICSI cycles using cryopreserved testicular sperm

ETC=embryo transfer cycles,

ET=number of transferred embryos

CP=clinical pregnancy

LBDR=live birth delivery rate.

Significant differences (*p*<0.05) between TESE/ICSI with fresh sperm and TESE/ICSI with cryopreserved sperm.

Concerning live births (Table 4), cycles with fresh spermatozoa yielded significantly higher live birth rates (65.2% *vs*. 23.8%, *p*=0.006). There were no significant differences regarding gestation age (*p*=0.444). Another healthy child was born after a frozen-thawed embryo transfer cycle, belonging to the fresh group (caesarean delivery at 39 weeks of gestation, female newborn). There were more female (70%) than male (30%) newborns. None of the newborns presented malformations; all had normal numerical chromosome constitutions for chromosomes 13, 18, 21, X and Y. Amniocentesis was performed in 3 cases and normal fetal karyotypes were confirmed. Early neonatal death (< 7 days after birth) occurred in the fresh testicular sperm group, apparently due to an abnormal insertion of the placenta (eutocic delivery at 36 weeks of gestation, male newborn, after transfer of 2 blastocysts, whose mother and father were aged 26 and 29 years, respectively). Almost a fifth (18.8%) of the 16 deliveries were preterm; 8.3% of them (36 weeks) were born from cycles with fresh spermatozoa and 50% (34 and 35 weeks) from cycles with cryopreserved spermatozoa. More than a third of the newborns (36.8%) had low birth weight; 35.7% of them were born from cycles with fresh spermatozoa, and 66.7% from cycles with cryopreserved spermatozoa.

**Table 4.** ICSI newborn outcomes of patients with non-mosaic Klinefelter syndrome using fresh and frozen-thawed testicular spermatozoa.

Parameters	TESE/ICSI		*p* value	Total
	Fresh sperm	Cryopreserved sperm		
Cycles (n)	25	22	-	47
Embryo transfer cycles (n)	23	2	-	44
Newborn (/ETC) (rate, n)	65.2 (15/23)	23.8 (5/21)	0.006	45.5 (20/44)
Male (/NB) (rate, n)	26.7 (4/15)	40 (2/5)	0.573	30 (6/20)
Female (/NB) (rate, n)	73.3 (11/15)	60 (3/5)	0.573	70 (14/20)
NB malformations (/NB) (rate, n)	0	0	-	0
EN death (/ETC) (n, rate) (≤ 7 days after birth)	4.3 (1/23)	0	-	2.3 (1/44)
Gestational age (mean ± SD, range)	37.9±1.7 (34-40)	37.0±2.9 (34-40)	0.444	37.7±2.0 (34-40)

TESE=testicular sperm extraction

ICSI=intracytoplasmic sperm injection

TESE/ICSI-fresh sperm=TESE/ICSI cycles using fresh testicular sperm

TESE/ICSI-cryopreserved sperm=TESE/ICSI cycles using cryopreserved testicular sperm

ETC=embryo transfer cycles, NB=newborn

EN death=early neonatal death.

Significant differences (*p*<0.05) between TESE/ICSI with fresh sperm and TESE/ICSI with cryopreserved sperm.

## DISCUSSION

The study population belongs to a special non-mosaic KS phenotype. These patients, besides infertility, did not show any signs or symptoms of testosterone deficiency. Most men had decreased mean testicular volume and their hormone profiles confirmed high mean serum levels of FSH and LH, with normal mean testosterone values in most of the cases. Patients with KS may present mild physical abnormalities, a possible explanation for why they do not receive clinical attention until they seek medical advice for infertility as adults (Bonomi *et al*., 2017). Our series gives further evidence of the clinical and hormonal heterogeneity of KS in patients visiting infertility clinics.

We did not find significant differences between patients with and without successful sperm retrieval (SSR) in terms of age, time of infertility, testicular volume, or serum hormone levels. Our results were similar to the ones published in previous studies (Madureira *et al*., 2014; Vicdan *et al*., 2016; Corona *et al*., 2017). However, other studies described higher sperm retrieval rates (SRR) associated with lower mean age (Okada *et al*., 2005a; Emre Bakircioglu *et al*., 2006; Ozer *et al*., 2018), lower FSH, and higher testosterone levels (Ozer *et al*., 2018).

Microsurgical TESE (Schlegel, 1999) was developed to yield higher SSR rates. Although exhibiting a variable range of success, several reports have evidenced higher SRR rates with microsurgical TESE (mTESE) than conventional TESE (cTESE) (Chen *et al*., 2019). However, some studies did not confirm higher SRR rates in mTESE (Guo *et al*., 2020). Comparisons between the two methods were recently reviewed. A meta-analysis involving 1248 KS patients showed no significant differences in SSR rates between the two methods, with a mean SRR rate of 44% (43% in cTESE, 45% in mTESE, 41% in mixed cases) (Corona *et al*., 2017). A more recent literature review including KS patients also reported SRR rates of 42-57% using both methods of testicular sperm retrieval (Chen *et al*., 2020). These differences stress the fact that SSR variability might not be due only to the different number of patients studied or the retrieval technique, but also to differences in patient characteristics. Along these lines, in our special group of KS patients the calculated SRR rate of 40.3% is comparable with previous reports using either cTESE or mTESE.

In the present report, there were 47 ICSI treatment cycles, 25 with fresh and 22 with cryopreserved testicular spermatozoa. No significant differences were observed between both groups in relation to the rates of oocyte maturity, embryo cleavage, high quality embryos and blastocyst formation. However, there were significantly higher fertilization, biochemical pregnancy, clinical pregnancy, implantation, and live birth rates in the fresh spermatozoa group.

Several previous studies reported the use of cryopreserved testicular spermatozoa (Ron-El *et al*., 2000a; Friedler *et al*., 2001; Bergère *et al*., 2002; Westlander *et al*., 2003; Okada *et al*., 2005b; Kyono *et al*., 2007; Vicdan *et al*., 2007; Greco *et al*., 2008), but they consisted of small case series or single case reports. Three recent studies provided larger numbers of patients and enabled clearer comparisons between fresh and cryopreserved spermatozoa treatment cycles. In a report with 38 KS patients with an SRR rate of 39.5% using cTESE and mTESE, 10 fresh and 16 cryopreserved testicular spermatozoa treatment cycles were performed. The authors observed a significant higher embryo cleavage rate in ICSI performed with cryopreserved testicular spermatozoa (Greco *et al*., 2013). In a previous report by our group including 65 KS patients with an SRR rate of 38.5% using cTESE, 20 fresh and 17 cryopreserved testicular spermatozoa treatment cycles were performed. The authors observed significantly higher rates of fertilization, high quality embryos, and clinical pregnancy in ICSI cycles using fresh testicular spermatozoa (Madureira *et al.*, 2014). In another report including 83 KS patients with an SRR rate of 42.1% using cTESE and mTESE, 32 fresh and 12 cryopreserved testicular spermatozoa treatment cycles were performed. The authors observed a significantly higher implantation rate in ICSI with cryopreserved testicular spermatozoa (Vicdan *et al*., 2016). In conclusion, two studies indicated that ICSI cycles with cryopreserved testicular spermatozoa were associated with higher rates of embryo cleavage and implantation, while two studies (including the result reported herein) found that ICSI cycles using fresh testicular spermatozoa were associated with higher fertilization, embryo cleavage, high quality embryo, implantation, pregnancy, and live birth rates.

These differences deserve some attention. First, in a meta-analysis including cases of non-obstructive azoospermia, the authors found no significant differences in embryological or clinical outcomes from ICSI using either cryopreserved or fresh testicular spermatozoa. However, the authors also observed that better outcomes were reached with fresh testicular sperm when different diseases were individually analyzed (Ohlander *et al*., 2014). Regarding the surprising observations that the use of cryopreserved testicular spermatozoa yielded higher embryo cleavage and implantation rates (Greco *et al*., 2013; Vicdan *et al*., 2016), since only two parameters were analyzed and the number of cases included was relatively low, data may indicate that in reality there are no critical clinical differences between ICSI outcomes either using fresh or cryopreserved testicular spermatozoa.

In relation to previous observations (Madureira *et al*., 2014) and findings of the present study that support better embryological and clinical outcomes with fresh testicular spermatozoa than with cryopreserved testicular spermatozoa, some hypotheses can be raised regarding the conclusion described in the meta-analysis in which no differences were found in embryological and clinical outcomes when fresh and cryopreserved testicular spermatozoa were compared (Ohlander *et al*., 2014). First, this meta-analysis included all types of non-obstructive azoospermia, and not only individuals with KS. Secondly, we may assume that the present results may be due to the greater number of cases studied with cryopreserved testicular spermatozoa and/or the specific KS population analyzed. Another hypothesis might be the lower quality of cryopreserved spermatozoa retrieved from diagnostic TESE. However, comparisons between TESE and diagnostic TESE revealed no differences between the two (data not shown), besides the fact that only five cycles were performed with cryopreserved spermatozoa from diagnostic TESE.

In KS patients, spermatozoa (Ferlin *et al*., 2005) and preimplantation embryos (Staessen *et al.,* 2003) exhibit a higher rate of autosome and sex chromosome aneuploidies. There are two reports with fetal reduction due to a 47,XXY karyotype after ART treatments (Ron-El *et al*., 2000b; Friedler *et al*., 2001). Nevertheless, all published data revealed that, with the exception of these two cases, none of the newborns from KS patients showed abnormal chromosomal constitutions (Madureira *et al*., 2014; Corona *et al*., 2017; 2019; Miki *et al*., 2017; Chen *et al*., 2020). This clearly reassures couples that men with KS are not at increased risk of transmitting their genetic condition to their offspring. The birth of healthy children from KS patients using testicular spermatozoa may be explained by observations that the majority of the 47,XXY germ cells are not meiotically competent. Additionally, studies also showed that a normal pattern of sex chromosome segregation occurs in most of the testicular spermatozoa retrieved from KS patients (Garcia-Quevedo *et al*., 2011; Miki *et al*., 2017). In the present study, 21 healthy children were born without numerical chromosomal abnormalities.

## CONCLUSION

The data described herein account for one of the largest series of azoospermic patients with non-mosaic Klinefelter syndrome studied to date and of Klinefelter syndrome patients treated with cryopreserved testicular spermatozoa. After conventional TESE, the spermatozoa recovery rate was 40.3%, which is similar to the rates presented in most recent literature. Analysis of the clinical outcomes revealed high pregnancy and live birth rates, with all 21 newborns presenting no malformations or chromosomal aneuploidies. These results are very encouraging for the reproductive prognosis of non-mosaic Klinefelter syndrome patients. Future studies with a greater number of cycles using cryopreserved testicular spermatozoa are needed in order to confirm the existence of better clinical outcomes with fresh testicular spermatozoa.
